# What is the role of the cerebellum in the pathophysiology of dystonia?

**DOI:** 10.1007/s00415-019-09344-7

**Published:** 2019-04-29

**Authors:** W. K. W. Fung, K. J. Peall

**Affiliations:** 1 Department of Neurology, Institute of Psychological Medicine and Clinical Neurosciences, Cardiff University, Cardiff, CF14 4XW UK; 2 Division of Psychological Medicine and Clinical Neurosciences, Neuroscience and Mental Health Research Institute, Cardiff University, Cardiff, UK

## Introduction

Dystonia is one of the most common forms of movement disorder. It is caused by co-contraction of antagonistic muscle groups leading to abnormal postures and pain. As with many movement disorders, dystonia has long been thought to be due to disrupted basal ganglia signalling, although there is increasing evidence that it is a network-based disorder with involvement of multiple brain regions. There is increasing evidence to suggest that the cerebellum may also play a critical role in giving rise to dystonia, with transgenic murine models of the DYT1-form of dystonia demonstrating aberrant cerebellar pathways. Furthermore, neuroimaging studies have demonstrated decreased grey matter volume in the cerebellum as well as increased resting state functional connectivity between the cerebellum and sensorimotor areas. In this month’s journal club, we will be discussing three recent publications that seek to better understand the role of the cerebellum in dystonic disorders.

## Abnormal cerebellar connectivity and plasticity in isolated cervical dystonia

Transcranial Magnetic Stimulation (TMS) is a non-invasive technique that involves application of a brief (< 1 ms) pulse of high-intensity magnetic field (2 T), enabling activation of neurons up to 5 cm below the scalp surface. If two areas of the brain are stimulated simultaneously, this is termed paired-pulse transcranial magnetic stimulation (ppTMS). Other forms of stimulation include repetitive TMS (rTMS): continuous Theta Burst Stimulation (cTBS), a 40-s train of uninterrupted TBS, in this case given over the right cerebellar hemisphere, and thought to be downregulating, and intermittent Theta Burst Stimulation (iTBS), a 2-s stimulation followed by an 8-s pause, considered to be an upregulating stimulus.

Twelve patients diagnosed with cervical dystonia (6 males, 6 females, mean age 47 years, disease duration 8 years) and thirteen control subjects were recruited. This ppTMS study was carried out over two different sessions, performed at least 1 week apart, in order to investigate the effects of repetitive stimulation. The effects of the cTBS and iTBS stimulation to the right lateral cerebellum was measured in terms of the excitability of the primary motor cortex (left M1), cerebellar cortical connectivity at rest, and cerebellar plasticity. Prior to the study, any oral medications were withheld for 48 h, and botulinum toxin injections were given at least 3 months previously. At each session, motor evoked potentials (MEPs), short intracortical inhibition/facilitation (SICI–ICF) (achieved through stimulation of inhibitory interneurons) and cerebellar brain inhibition (CBI) of the primary motor cortex were recorded at baseline and following rTMS, at time 0, after 20 min and 40 min (t0, t20 and t40).

SICI–ICF results demonstrated that the conditioning stimulus was able to inhibit the MEP amplitude in the control group only; at the 5-ms inter-stimulus interval (ISI), MEP amplitude in controls was 0.75 of test, while in dystonic patients this was higher at 1.01 (*p* = 0.009). Patients with cervical dystonia did not show cerebellar inhibition at baseline; in fact the data demonstrated a trend in which more inhibition (lower CBI values) was associated with lower clinical severity scale scores. Cerebellar iTBS demonstrated a facilitation of MEP amplitude from baseline while cTBS resulted in MEP inhibition in the control group only (*p* = 0.02 and *p* = 0.029, respectively). Both iTBS and cTBS protocols failed to facilitate or inhibit MEP amplitudes in dystonic patients.

**Comment:** The absence of cerebellar inhibition at baseline, and no significant change after repetitive TMS stimulation, suggests a disruption to normal cerebellar plasticity in those diagnosed with dystonia. Although the results from this study demonstrate clear distinctions between those with and without dystonia, the results are limited by stimulation of a body part unaffected by dystonia (first dorsal interosseus muscle), the small numbers within each cohort and the absence of a sham group for comparison.

Porcacchia P. et al. PLoS One. 2019 Jan 25;14(1):e0211367.

## Abnormal cerebellar processing of the neck proprioceptive information drives dysfunctions in cervical dystonia

Paired associative stimulation (PAS) refers to a model consisting of slow-rate repetitive low-frequency median nerve stimulation combined with TMS over the contralateral motor cortex. Contrary to previous studies showing that excitation of the cerebellar cortex can block increased motor cortical excitability induced by PAS, this study involving 22 individuals diagnosed with cervical dystonia demonstrated the opposite findings. Here, cerebellar inhibition suppressed the effects of PAS, while cerebellar excitation enhanced them. A second experiment involving two groups of healthy subjects (*n* = 12) demonstrated that head turning was sufficient to invert the cerebellar modulation of motor cortical plasticity, suggesting this was not feature-specific to cervical dystonia. Finally, further evaluation, also involving healthy control subjects (*n* = 6), also showed that proprioceptive perturbation (by vibration) of the left sternocleidomastoid (SCM) muscle had the same effects as turning the head.

In order to investigate cortical plasticity, PAS was carried out after the cerebellar output was modulated by decreasing or increasing the cerebellar cortex excitability by repetitive transcranial magnetic stimulation [cTBS or iTBS protocols, or sham stimulation (Sham_CB_)]. In healthy subjects maintaining their head straight in a neutral position, cTBS over the lateral cerebellum enhanced the contralateral motor cortical responsiveness to paired associative stimulation, while iTBS over the lateral cerebellum blocked it entirely, confirming previous reports. In the neck rotation cohort (individuals with cervical dystonia, healthy controls with head rotation and during SCM vibration), cTBS_CB_ and iTBS_CB_ had opposing effects on PAS: cTBS_CB_ blocked the increase in contralateral motor cortical activity responsiveness to PAS, while iTBS_CB_ resulted in enhancement.

**Comment:** These results are the first to demonstrate that proprioceptive feedback from the SCM plays a potential role in modulating how the cerebellum impacts the plasticity of the primary motor cortex. This study suggests that abnormal cerebellar processing of the neck proprioceptive information drives dysfunction of a potential midbrain neural integrator, previously proposed to be located within the interstitial nucleus of Cajal (INC) in cervical dystonia, resulting in head turning and maintaining a cycle of continuous muscle activity. Further work is required to determine a more comprehensive understanding of a neural integrator role, and whether sensory input such as the ‘geste antagoniste’ impacts this outcome.

T. Popa. et al. Sci Rep. 2018 Feb 2;8(1):2263.

## Oscillatory cortical activity in an animal model of dystonia caused by cerebellar dysfunction

This study used a murine model to investigate the contribution of the cerebellum to oscillatory brain activity in dystonia. Twenty 3-month-old Swiss albino mice were split into two groups: (1) motor-somatosensory group (*n* = 10), and (2) motor-parietal group (*n* = 10), dependent on the placement location of the cortical electrode. Microinjections of kainic acid were administered to the cerebellar vermis to induce dystonic movements and compared to baseline when no injections were given. Measurements included: somatosensory, motor and parietal cortical oscillatory activity and connectivity during the dystonic episodes; electromyographical (EMG) recordings of the neck muscles; and electrocorticograms (ECoG) measured 10 min prior- and 150 min after the kainic acid injections. The movement of the mice was measured using video tracking, with dystonia severity assessed by four reviewers blinded to the procedures.

The results demonstrated that application of kainic acid generated reproducible dystonic motor behaviour. Average dystonia scores reached a peak at 50 min post-injection, with highest dystonia severity scores seen on day 3, and lowest on day 5 of injections. EMG recordings demonstrated significantly increased median frequency of muscular power spectral density (PSD) on days 1, 4 and 5 (*p* < 0.001, *p* < 0.01 and *p* < 0.05, respectively), indicating that kainic acid continued to exert a muscular effect in spite of decreasing dystonia severity scores. There was also a decrease in coherence of motor-ipsilateral somatosensory and motor-ipsilateral parietal cortices in all frequency bands both pre- and post-kainate treatment, suggesting a diffuse reduced functional connectivity between the cortices. Finally, recordings also demonstrated an increase in somato-sensory and parietal cortex ECoG PSD, especially on days 4 and 5 for both pre- and post-kainate recordings, suggesting increased neural activation and, therefore, abnormal processing of multisensory input as well as abnormal activation of the parietal cortex, which also can be observed in dystonia.

**Comment:** Overall, this study showed that cerebellar stimulation is able to alter neuronal activity across the cortical sensorimotor/parietal networks. The reduction in coherence between these areas suggests that synchrony can be modulated by the cerebello-cortical network. As described in the paper, the combination of an increase in gamma band and active wake behaviour in the pre-kainate state, together with a decrease in the average dystonia score on days 4 and 5, suggests a possible adaptive process to correct the dystonic movements. Although interesting, these functional phenotypic findings must be considered in the context of a murine model, and may not directly relate to the pathophysiological underpinnings of dystonia in humans.

Georgescu E.L.. et al. Front Cell Neurosci. 2018 Nov 6;12:390.

